# Application of unicompartmental knee arthroplasty in the treatment of knee osteoarthritis

**DOI:** 10.1186/s42836-020-00065-1

**Published:** 2021-02-15

**Authors:** Bing Wang, Haining Sun, Zhihou Fu, Jian Kang, Xiuchun Yu

**Affiliations:** Department of Orthopedic, The 960 Hospital of PLA, 250031 Jinan, China

**Keywords:** Arthroplasty, Replacement, knee, Unicompartmental knee arthroplasty, Long term adverse effects

## Abstract

**Objective:**

For the treatment of medial compartment knee osteoarthritis, unicompartmental knee arthroplasty was chosen on the basis of the clinical effects and the rate of prosthesis survival. A retrospective analysis was performed on 500 patients with osteoarthritis of the medial compartment knee treated by unicompartmental knee arthroplasty between April 2007 and April 2017. The cohort was comprised of 176 males and 324 females, aged (61.12 ± 8.0) years old. The clinical treatment was evaluated in terms of the range of movement (ROM), the Knee Society score (KSS), and the Oxford knee score.

**Results:**

The follow-up lasted 1.59–11.60 years. Grouped in terms of age, 133 cases were in the middle-age, 295 were in the old-age group, and 72 in the advanced-age group. Against the Iwano classification of the patellofemoral joint, 104 cases were graded 0–1; 179 were graded 2; 182 were graded 3 and 35 were graded 4. The KSS score improved from (59 ± 5.6) points before the operation to (93 ± 3.5) points after the operation. The OKS score increased from (24 ± 2.4) points before the operation to (45 ± 3.8) points after the operation. The ROM of knee joint was (111 ± 6.8)° before the operation and was (117 ± 9.7)° after the operation. The 10-year survival rate of the prosthesis was 96%.

**Conclusion:**

UKA is one of the treatments for medial compartmental knee osteoarthritis. The issues, such as age and patellofemoral joint degeneration, can be addressed by careful selection of patients and precise operational manipulation.

## Background

Osteoarthritis (OA) of the knee represents one of the most common diseases among middle-aged and elderly people, and tends to involve the medial compartment of the knee joint [1]. Since McKeever [2] first proposed the concept of unicompartmental knee arthroplasty (UKA) in 1955, the metal prosthesis of unilateral tibial plateau has been inserted into the diseased joint compartment to treat OA. Initially, UKA had a high failure rate [3, 4]. Since 1970s, Goodfellow et al. designed the Oxford unicompartmental knee prosthesis, which consists of a spherical femoral, a flat tibial, and a mobile meniscal bearing [5–7]. In recent years, the Oxford UKA (OUKA) exhibited a clinical effect similar to that of total knee arthroplasty [8–11].

The OUKA only involves the single compartment of the knee and retains the bone mass and ligament function of the joint. It goes well with minimally invasive incision and restores the knee joint function early and quickly [12]. Some clinical studies have confirmed that UKA reduces pain and improves the range of motion (ROM) and joint function of the knee, with significant short- and medium-term clinical efficacy [13, 14]. The unconstrained design of OUKA allows rotation and translation, which ensures a large contact area of the prosthesis and reduces the stress between the prostheses, thereby minimizing the wear and prolonging the survival time. Price and Svard et al. reported that the 10-, 15-, and 20-year survival rates of OUKA prosthesis were 95%, 93.1%, and 91%, respectively [15–17]. Pandit et al. reported a 10-year survival rate of 94% and a 15-year survival rate of 91% in 1000 OUKA prostheses [18, 19].

However, the indications, complications, and prosthesis revision rate of the OUKA remain controversial [20–23]. The selection of the patients is a major factor affecting the survival rate of the prosthesis [24–26]. In 1989, Kozinn and Scott [27], for the first time, proposed the surgical indications for the UKA. Although these indications lack support of sufficient clinical data, they are currently in use. They include: (1) OA or osteonecrosis is confined to one compartment; (2) Low activity requirements; (3) Body weight < 82 kg; (4) Age > 60-year-old; (5) Slight pain in the affected knee joint at rest; (6) Scope of the knee joint flexion and extension > 90°; (7) Flexion contracture deformity < 5°; (8) Inside the knee, valgus < 15° and can be corrected. The indications for this operation are conservative. With the continuous improvement in the design of prosthesis and the perfection of the surgical techniques, the aforementioned indications can be expanded further. This retrospective study analyzed the long-term follow-up results of 500 patients with KO treated by OUKA and explored the indications, clinical effect, and prosthesis survival rate of OUKA.

## Materials and methods

Inclusion criteria: (1) The patients were diagnosed as having osteoarthritis of knee; (2) Preoperative imaging confirmed the onset of medial compartmental osteoarthritis of the knee (Ahlback radiographic investigation, grade 2–3 [28]), with the whole cartilage of lateral compartment still present, and the degree of patellofemoral joint degeneration was not taken as the evaluation standard; (3) Painful medial compartmental osteoarthritis without prepatellar pain or with mild prepatellar pain; (4) Anterior cruciate ligament and medial collateral ligament function was intact, allowing partial injury of the ligament surface; (5) The flexion deformity of the knee joint was < 15°, varus deformity was allowed but must be corrected for knee flexion; (6) Age and weight were not used as the selection criteria. The aforementioned criteria were similar to those proposed by the OUKA prosthesis design team when they reported the long-term survival of patients with a prosthesis [7].

Exclusion criteria: (1) Concurrently having other joint diseases, such as rheumatoid arthritis, joint infection, joint tuberculosis, bone tumor, and acute phase of intra-articular fracture; (2) Having degeneration of the lateral compartment of the knee joint or with concurrent severe deformity of the knee joint; (3) Patients had received ipsilateral knee osteotomy or joint replacement; (4) Patients could not tolerate surgery.

From April 2007 to April 2017, a total of 522 patients with knee osteoarthritis were treated with OUKA. According to the above-mentioned criteria, 500 cases of UKA surgery were included in this study. The third generation of Oxford mobile-bearing prosthesis system (Biomet Ltd., Bridgend, UK) was selected for all patients. The cohort was comprised of 176 (35.2%) males and 324 (64.8%) females, with the average age being (61.12 ± 8.0) years (range, 43–91). The patients were divided into three groups according to the age: a middle age group (< 55 years old), an old-age group (55–70 years old), and an advanced-age group (> 70 years-old). The body mass index (BMI) was (23.7 ± 4.2) kg/m^2 ^( range, 19.3–35.6 kg/m^2^). The unilateral replacement was performed in 400 cases (80.0%), including 205 cases of the right knee (41.0%) and 195 cases of the left knee (39.0%). The bilateral replacement was done in 88 cases (17.6%). One side received OUKA and the other side received TKA in 12 cases (2.4%). Iwano et al. [29] proposed a 0–4 scale for imaging assessment of patellofemoral joint degeneration on the basis of the axial X-ray of the patellofemoral joint (Grade 1: Joint-space narrowing is mild, in which the joint space is > 3mm. Grade 2: Joint-space narrowing is moderate, in which the joint space is < 3mm, but there is no bony contact. Grade 3: Joint-space narrowing is severe, in which the bone on bone area is < 1/4 of the joint surface. Grade 4: Joint-space narrowing is severe, the joint bony surfaces touch each other).

In all patients, operation was performed by the same group of surgeons. These patients were reviewed by a non-surgical medical team 1 month, 3 months, and 1 year after operation. The ROM, the Knee Society score (KSS) [30], and Oxford knee score (OKS) [31] were recorded to evaluate the clinical effect. All complications following surgery were recorded.

Descriptive statistics were used for data analysis. Kaplan-Meier survival analysis was employed for the evaluation of the prosthesis survival rate at different ages and the degree of patellofemoral joint degeneration. The revision of prosthesis for any reason was used as the endpoint, including replacement or removal of any component of the prosthesis. Two-sided independent sample *t*-test was utilized to compare the pre- or postoperative ROM, KSS, OKS. One-way analysis of variance (ANOVA) was used to compare postoperative ROM, KSS, and OKS between different age groups and patellofemoral joint degeneration groups. A *p* < 0.05 indicated a statistically significant difference. Statistical analysis was performed using SPSS statistical software (version 19.0, SPSS Inc., IBM Co., USA).

## Results

The 500 patients recruited in this study were followed up for 1.59–11.60 years (average: 5.27 years). The last follow-up was in November 2018. In terms of age, 133 (26.6%) cases were in the middle-age group, 295 (59.0%) in the old-age group, and 72 (14.4%) in the advanced-age group. Against the Iwano imaging classification, 104 cases (20.8%) were rated 0–1; 179 cases (35.8%) were rated 2, 182 cases (36.4%) were rated 3 and 35 (7.0%) were rated 4. The KSS score was improved from (59 ± 5.6) preoperatively to (93 ± 3.5) postoperatively (*t*=–2.586, *p* < 0.05). The OKS score increased from (24 ± 2.4) preoperatively to (45 ± 3.8) postoperatively (*t*=–2.056, *p* < 0.05). The average preoperative ROM of the knee joint was (111 ± 6.8) °, and the postoperative ROM was (117 ± 9.7)° (*t*=–2.334, *p* < 0.05).

**Fig. 1:**
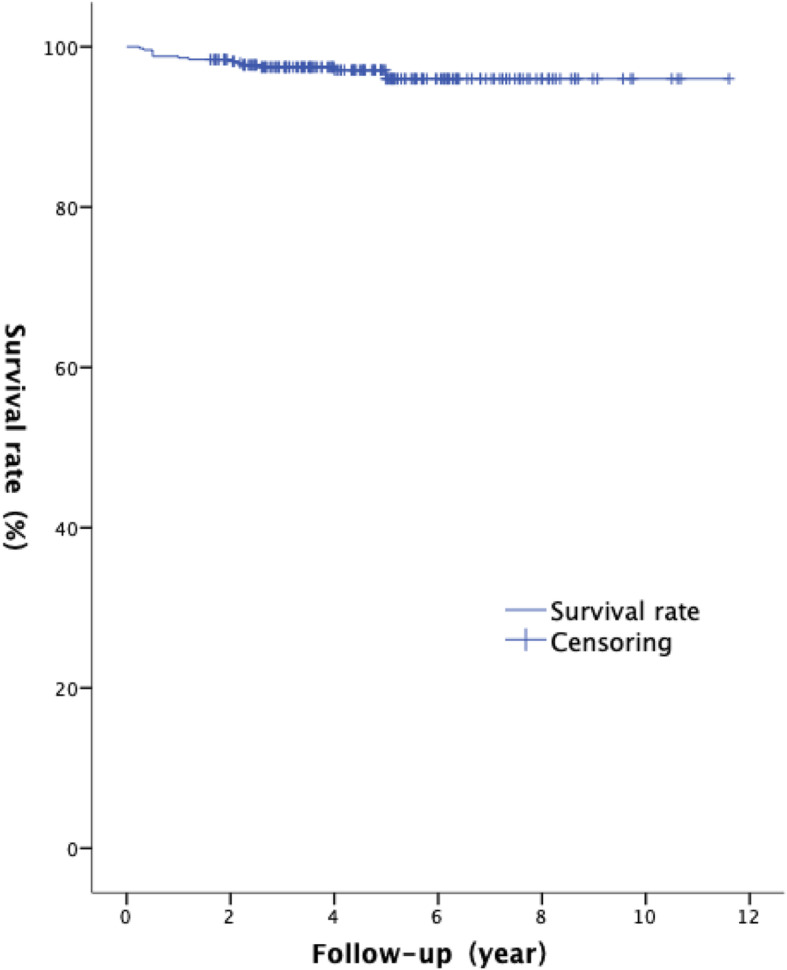
Kaplan-Meier survival analysis, 10-year survival rate is 96%

The 10-year survival rate of the prosthesis was 96.0% (Fig. 1). Among the 500 patients, 20 (4%) developed complications, and 12 (2.4%) had the highest bearing dislocation rate. Of the 12 cases of bearing dislocation, 6 was due to the excessive flexion of the knee caused by injury, 3 due to the severe wear of bearing, 2 due to aseptic loosening of the femoral component, and 1 due to postoperative laxity of the anterior cruciate ligament. Furthermore, 9 of the 12 bearing dislocation cases were treated with replacement of the thick bearing, and 2 cases of loosening femoral component were treated with femoral component revision. In addition, the one case of function loss of anterior cruciate ligament was treated with a total knee prosthesis revision. The postoperative dislocation of the bearing occurred between 0.25 and 5 (average, 2.1) years.

Other complications included delayed deep infection in 1 patient (0.2%) and the infection developed 2 years after surgery and was treated by cement spacer and staged total knee arthroplasty. Moreover, 4 cases (0.8%) had loosening of the femoral component of the prosthesis (including 2 cases of bearing dislocation), of which 3 underwent revision of femoral component and 1 received total knee arthroplasty. In 2 cases, prosthesis loosening resulted from spontaneous osteonecrosis of the knee (SONK). Two patients (0.4%) developed repeated aseptic hydrops in the joint and recovered after knee puncture and physical therapy. 2 cases (0.4%) had a bony free body from the osteophyte in the joint, which was removed by arthroscopic surgery, and 1 patient (0.2%) suffered from postoperative knee joint pain of unknown origin, which subsided after conservative treatment and rehabilitation exercise.

**Fig. 2:**
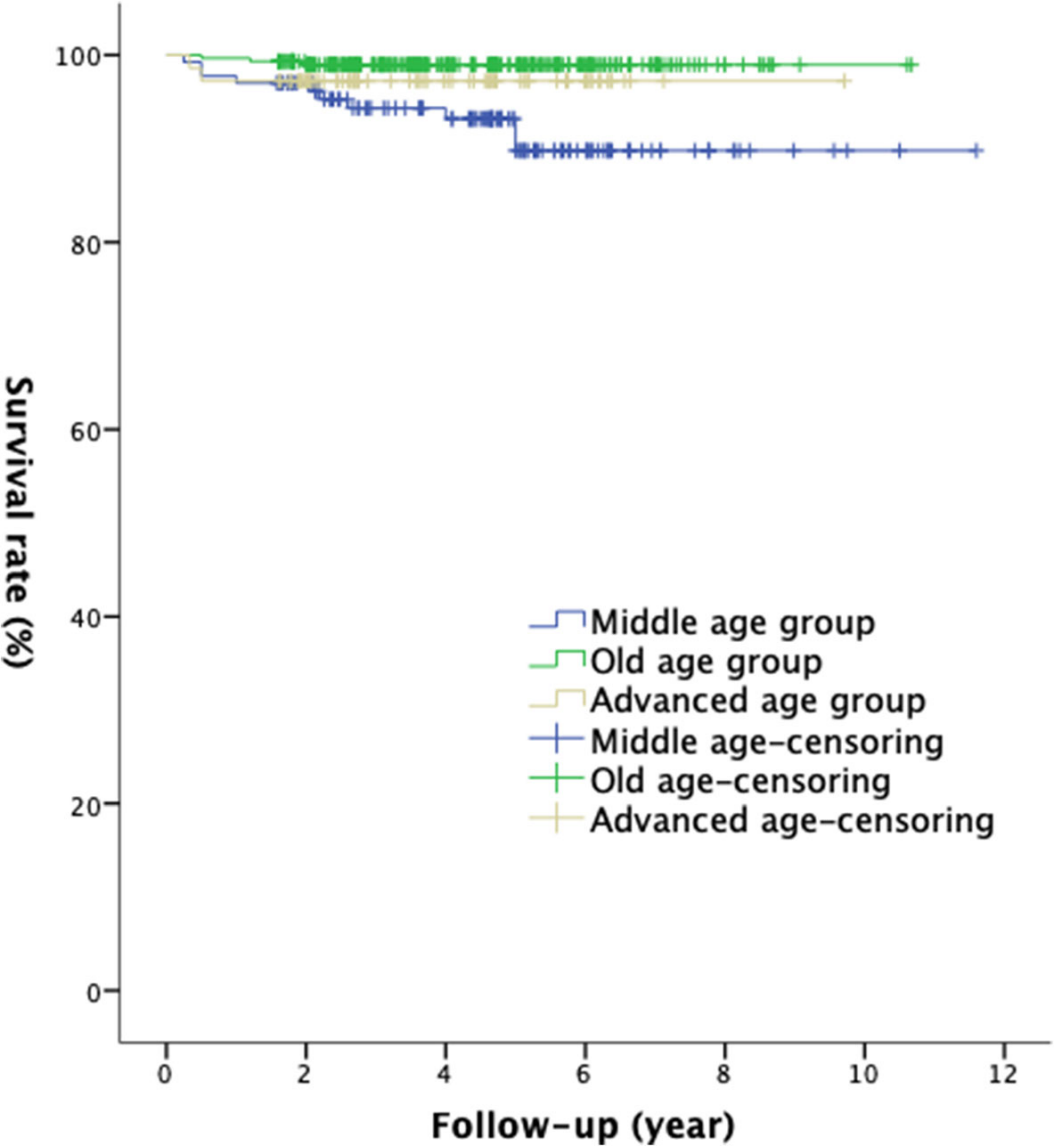
Kaplan-Meier survival analysis of different age groups: the 10-year survival rate in the middle-age group was 89.8%, that of the advanced-age group was 99.0%, and that of the older age group was 97.2% (*χ*^2^ = 12.37, *p* = 0.02)

The 10-year prosthesis survival rate was 89.8% in the middle-age group (*n* = 133), 99.0% in the old-age group (*n* = 295), and 97.2% in the advanced-age group (*n* = 72). The long-term survival rate of the prosthesis was significantly reduced in patients < 55-year old (*χ*^2^ = 12.37, *p* = 0.02) (Fig. 2). However, no significant difference was found in the postoperative KSS and OKS scores among different age groups, but the postoperative ROM of the knee was significantly greater in the old-age group than in the advanced-age group (*F* = 3.049, *p* = 0.048).

**Fig. 3:**
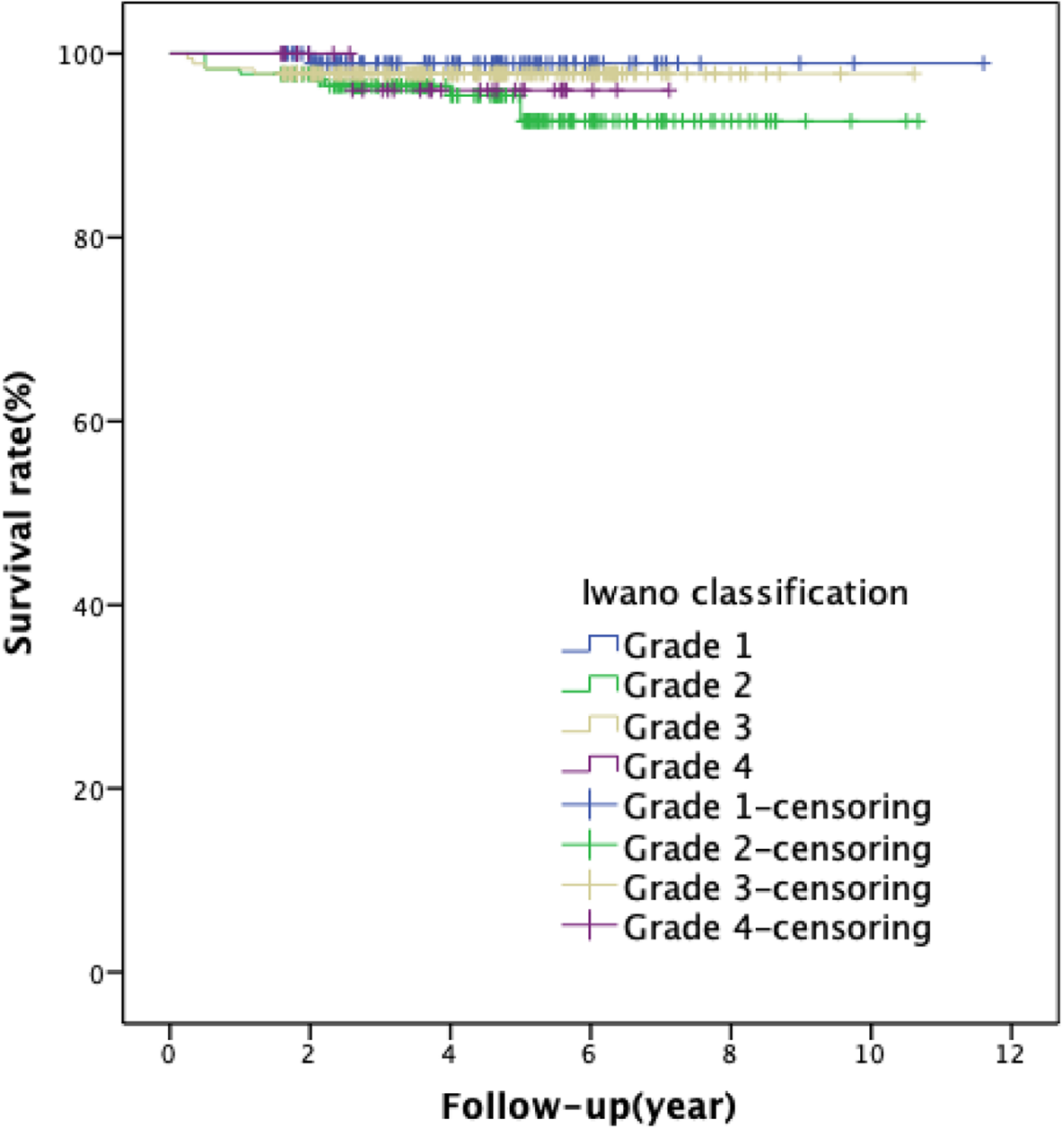
Kaplan-Meier survival analysis for Iwano classification: the 10-year survival rate of grade 0–1 group was 98.9%, that of grade 2 group was 92.7%, that of grade 3 group was 97.8%, and that of grade 4 group was 96% (*χ*^2^ = 4.162, *p* = 0.244)

According to the Iwano imaging classification, the 10-year prosthesis survival rate was 98.9% in the grade 0–1 group, 92.7% in the grade 2 group, 97.8% in the grade 3 group, and 96% in the grade 4 group. Interestingly, the degree of patellofemoral joint degeneration did not affect the survival rate of the UKA (*χ*^2^ = 4.162, *p* = 0.244) (Fig. 3). In terms of clinical efficacy, although no significant difference existed in the postoperative ROM and KSS scores across all groups, the degree of patellofemoral joint degeneration did not exert a significant impact on the postoperative OKS scores (*F* = 10.627, *p* < 0.001), but was significantly reduced in the grade 4 group.

## Discussion

This study reviewed 500 OUKAs for the treatment of medial compartment osteoarthritis of the knee in a single institution. The results showed that OUKA significantly improved knee joint mobility, and could achieve reliable clinical effect. Thus, OUKA is the best choice for the treatment of anteromedial osteoarthritis. Murray et al. first reported that the 10-year survival rate of the phase III Oxford UKA was 98% [7]. In this study, the 10-year survival rate of the prosthesis in 500 patients was 96%, which was consistent to recent studies [18, 32, 33]. The long-term survival rate of OUKA did not differ significantly between the consultant and trainee surgeons. However, the postoperative complications occurred more with the trainee surgeons. The operation needed some learning curve and experience[34]. The surgical technique and clinical experience are the key factors to the improved the clinical effect and reduced complications.

The complications of OUKA include: polyethylene bearing dislocation, bearing wear, aseptic loosening, medial collateral ligament or cruciate ligament injury, arthritis progression, deep infection, articular cavity recurrent hematoma, bone cement residual, and unexplained pain. The complications varied with different periods of follow-up [35, 36]. The dislocation of bearing is one of the main postoperative complications. The incidence of dislocation is higher in Asia than in other regions, which could be attributed to the inadequate stability of the bearing in high flexion, which was frequent in Asian patients [36, 37]. In Europe and USA, the progression of lateral compartment osteoarthritis is the most common complication (4.2%) [38]. In our cohort, 4 patients developed aseptic loosening of the femoral prosthesis, including 2 cases of spontaneous osteonecrosis of the knee (SONK), which occurred due to postoperative bone absorption in the necrotic area. The related clinical studies showed that SONK could be addressed by OUKA with satisfactory clinical effect [39, 40] but further multicenter randomized controlled studies are warranted to confirm the findings. Thus, SONK should be applied with caution.

The complications can be treated with revision surgery or non-revision surgery [41, 42]. Non-revision surgery includes debridement, arthroscopic surgery, and ligament reconstruction. The revision surgery includes simple bearing replacement, UKA prosthesis revision, or total knee prosthesis revision, depending on the type of complications. The overall incidence of complications is not high with appropriate selection of patients and skillful surgical procedure.

For advanced-age patients, especially those > 75-year-old, the OUKA has the apparent advantages of small trauma, fast postoperative recovery, reliable clinical efficacy, and high long-term survival rate of the prosthesis, as compared with TKA [43]. Although the postoperative range of motion of the knee in the advanced-age patients was marginally lower than that of the other two groups, the clinical efficacy and long-term survival rate of the prosthesis were essentially identical. Nonetheless, the use of OUKA in younger and physically-active patients remains controversial. According to the records of joint registration systems in some countries [24, 26], patients < 65-year-old often have poor postoperative outcomes and high revision rates. Nevertheless, some single-center studies showed that younger and more active patients who had undergone UKA surgery attained satisfactory clinical efficacy, and they were able to return to normal activity, could engage in sports, and presented a high long-term survival rate [44, 45]. In this study, the 10-year survival rate of the prosthesis in the middle-age group was only 89.8%, which was lower than that of the other two groups. However, the postoperative clinical efficacy was satisfactory, and the clinical score was significantly improved, but no difference was found as compared to the other groups. Thus, accurate prosthesis placement is essential for stable and adequate postoperative clinical efficacy.

Patellofemoral joint degeneration in the past has been regarded as a contraindication of UKA [27]. However, a number of recent studies demonstrated that it had no influence on the postoperative effect. Therefore, some investigators began to abandon the previous indication. As the asymptomatic patients with patellofemoral joint degeneration are indicated for OUKA, some scholars ignored the patellofemoral joint degeneration [7, 46, 47]. In this study, patellofemoral joint degeneration did not affect the survival rate of OUKA. Iwano classification makes it simple and feasible to assess the degree of patellofemoral joint degeneration. In the patients with grade 4 degeneration, the layer of the cartilage of the lateral patellofemoral joint completely wore off, with bone-on-bone contact. The postoperative effect in such patients is poor. Hence, the patients’condtion should be carefully evaluated to achieve optimal results.

## Conclusions

In conclusion, OUKA is one of the treatments for medial compartmental osteoarthritis and has reliable clinical efficacy, high long-term survival rate, and low incidence of related complications. Some issues, such as age-related problems and patellofemoral joint degeneration, can be addressed by careful patient selection and skillful operation.

## Data Availability

The datasets used during the current study are available from the corresponding author upon reasonable request.

## Abbreviations

ROM: The range of movement
KSS: The Knee Society score
OKS: The Oxford knee score
UKA: Unicompartmental knee arthroplasty
OA: Osteoarthritis
OUKA: The Oxford Unicompartmental Knee Arthroplasty
KO: Knee osteoarthritis
BMI: The body mass index
SONK: The spontaneous osteonecrosis of the knee
